# Imported Dengue Cases, Weather Variation and Autochthonous Dengue Incidence in Cairns, Australia

**DOI:** 10.1371/journal.pone.0081887

**Published:** 2013-12-13

**Authors:** Xiaodong Huang, Gail Williams, Archie C. A. Clements, Wenbiao Hu

**Affiliations:** 1 School of Population Health, the University of Queensland, Brisbane, Queensland, Australia; 2 School of Public Health and Social Work, Institute of Health and Biomedical Innovation, Queensland University of Technology, Australia; Menzies School of Health Research, Australia

## Abstract

**Background:**

Dengue fever (DF) outbreaks often arise from imported DF cases in Cairns, Australia. Few studies have incorporated imported DF cases in the estimation of the relationship between weather variability and incidence of autochthonous DF. The study aimed to examine the impact of weather variability on autochthonous DF infection after accounting for imported DF cases and then to explore the possibility of developing an empirical forecast system.

**Methodology/principal finds:**

Data on weather variables, notified DF cases (including those acquired locally and overseas), and population size in Cairns were supplied by the Australian Bureau of Meteorology, Queensland Health, and Australian Bureau of Statistics. A time-series negative-binomial hurdle model was used to assess the effects of imported DF cases and weather variability on autochthonous DF incidence. Our results showed that monthly autochthonous DF incidences were significantly associated with monthly imported DF cases (Relative Risk (RR):1.52; 95% confidence interval (CI): 1.01–2.28), monthly minimum temperature (^o^C) (RR: 2.28; 95% CI: 1.77–2.93), monthly relative humidity (%) (RR: 1.21; 95% CI: 1.06–1.37), monthly rainfall (mm) (RR: 0.50; 95% CI: 0.31–0.81) and monthly standard deviation of daily relative humidity (%) (RR: 1.27; 95% CI: 1.08–1.50). In the zero hurdle component, the occurrence of monthly autochthonous DF cases was significantly associated with monthly minimum temperature (Odds Ratio (OR): 1.64; 95% CI: 1.01–2.67).

**Conclusions/significance:**

Our research suggested that incidences of monthly autochthonous DF were strongly positively associated with monthly imported DF cases, local minimum temperature and inter-month relative humidity variability in Cairns. Moreover, DF outbreak in Cairns was driven by imported DF cases only under favourable seasons and weather conditions in the study.

## Introduction

The geographical distribution of DF across the world has rapidly expanded in recent decades [1], [2]. Globalization and modern lifestyle changes have increased the number of international travellers around the world. Importation of DF cases has become an important means by which the dengue virus (DENV) spreads to local *Aedes aegypti* and *Aedes albopictus* mosquitoes, particularly in currently non-endemic countries [3], [4]. Although DF is not naturally endemic in Australia, the DF mosquito *Ae. aegypti* is common in northern Queensland and outbreaks can occur when the DENV is transmitted to the local mosquito population by infected international travellers [5]. Cairns experiences the highest number of DF cases in 2009 in Australia. Reappearance occurred in 1981 after a 26 year absence in Australia [6]. A significant increase has been noted in the number of imported DF cases and the frequency of outbreaks since the international airport was opened in 1984 in Cairns [7]–[9]. Annual outbreaks occur when imported DF cases infect the local *Ae. aegypti* mosquito population, setting up autochthonous DENV transmission [5]. Therefore, it is important to evaluate the effect of numbers of imported DF cases on the incidence of autochthonous DF in Cairns, Australia [10].

DENV is transmitted by mosquitoes of the genus *Ae. aegypti* and a number of previous studies have demonstrated that weather conditions can strongly influence breeding, maturation period, virus replication, density and life cycle of *Ae. aegypti* mosquitoes [11], [12]. DENV transmission is thus highly dependent on weather factors, including temperature, rainfall and relative humidity [13]–[15]. There is growing evidence that climate change may impact on DF emergence [11], [13], [16]–[18]. Recent studies have indicated that climate change does not just increase temperature but also increases the frequency and intensity of extreme weather events such as heatwaves, hurricanes and tropical cyclones, and drought [19]. However, uncertainty regarding the impacts of extreme weather events on DF incidence still exists and there is a need for further investigations [14].

The current study aimed to examine the relationship between the numbers of imported DF cases, weather factors, inter-month climate variations and autochthonous DF incidence, and to facilitate our understanding of the potential effects of imported DF cases and weather variability events on autochthonous DF incidence in Cairns.

## Materials and Methods

### Study Site and Data Collection

The study site was located at Cairns (latitude −16.55, longitude 145.46), a tropical city that is popular for domestic and foreign tourists, in the north of Queensland, Australia. The highest mean temperature (31°C) occurs in the period of December, January and February. The lowest mean temperature (17–18°C) is observed in the period of June, July and August. The monthly total rainfall is between 279 and 455 mm [20], making Cairns one of the wettest cities in Australia. The population size was 150,920 in the 2010 Census [21], making it the second largest Australian city (after Townsville) in the north of Queensland.

The data on numbers of notified DF cases in Cairns (the area based on Cairns Public Health Unit, which include Port Douglas and Innisfail areas). The data included both autochthonous DF cases and imported DF cases between 1^st^ January 2000 and 31^st^ December 2009 (Note, cases with an unknown country of origin were removed), which were provided by Queensland Health (the Queensland state government department of health). DF cases were confirmed by positive test results based on DF NS1 antigen, IgM antibody and PCR tests from clinical laboratories [5]. As DF is a notifiable disease, it is a legal requirement that positive test results are reported by laboratories to the Queensland Health, where data are archived by the Communicable Diseases Unit. Imported DF cases were defined as confirmed DF cases with a travel history to DF endemic countries within 12 days. Autochthonous DF cases had no travel history [5]. Daily climate data (including daily minimum temperature (MT), daily relative humidity (RH) and daily rainfall) were obtained at Cairns Aero from the Australian Bureau of Meteorology between 1^st^ January 2000 and 31^st^ December 2009. Monthly standard deviations were calculated for daily MT, daily rainfall and daily RH in each month for providing information on within-month climate variation. Annual population size in Cairns was obtained from Australian Bureau of Statistics.

### Statistical Analysis and Modelling

Generalised linear regression models with Poisson and negative binomial link have been widely applied to count data analysis in epidemiology. Count data often display over-dispersion due to excess zeroes and/or unobserved heterogeneity [22]. Poisson regression models entail a key assumption that the variance of the outcome is equal to its mean, and thus are not suited to the over-dispersed count data. The classical negative binomial model, which allows the variance to be greater than the mean, is typically used to account for over-dispersion. However, the negative binomial model lacks the ability to account for an excess in zero counts [23]. To account for excess zeroes, hurdle models and zero-inflated models have been commonly used [23]–[28]. The zero-inflated model treats excess zeroes as “structural” and allows for additional “sampling” zeroes, whereas the hurdle model only accommodates excess sampling zeroes. Some research showed that zero-inflated and hurdle models often perform similarly [23], [29]. Model choice between zero-inflated and hurdle models may be more dependent on the study objective or sampling design [23].

The monthly DF data exhibited both over-dispersion and excess zeroes. The hurdle model is more appropriate for data with too many zeroes and scarce cases [30]. Here, a time-series negative-binomial hurdle model (TNBH) was chosen in this case study. A hurdle model is composed of two parts: a binary response model generating the zero cases and a truncated-at-zero count model generating positive counts [31]. A hurdle model is given by
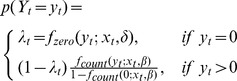
where *y_t_* represents the number of autochthonous DF cases at time period *t*; *β* and *δ* are the model regression coefficients; *f_count_* represents the count process with a negative binomial density; *f_zero_* corresponds to the hurdle part using a logistic regression. Let *u_t_* be the mean of the autochthonous DF count distribution. In this study, *u_t_* and *λ_t_* can be expressed as:



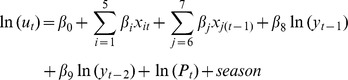


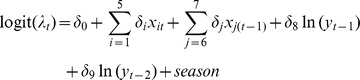
where *x_it_* represents *i*th independent variable at time period *t*, including monthly imported DF cases, monthly MT, monthly rainfall, monthly RH and monthly standard deviation for daily RH (SDRH); *x_6_*_(*t−*1)_ and *x_7_*_(*t*−1)_ represent monthly standard deviations of daily MT (SDMT) and daily rainfall (SDR) at a lag of 1 month, respectively. ln(*y_t−1_*) and ln(*y_t−2_*) are autoregressive terms at lag of 1 and 2 months (AR(1) and AR(2)), which deal with autocorrelation of the residuals. *P_t_* is population size at time period *t*. Adjustment was made for the population size by including an offset term ln(*P_t_*). The season term is a categorical variable (wet season: November–April), dry season: May–October) in order to adjust for the seasonal pattern. Log-transformations were required for the number of imported cases, lagged autochthonous DF cases, rainfall and SDR to normalize these variables, which were highly skewed.

Several statistics were calculated to assess goodness of fit of the model. The Vuong non-nested test was used to compare the TNBH model with a negative binomial model [32]. We also compared the TNBH with a Poisson model, a negative binomial model and a Poisson hurdle model using the Akaike Information Criterion (AIC) [33].

## Results

### Descriptive Analysis

**Figure 1:**
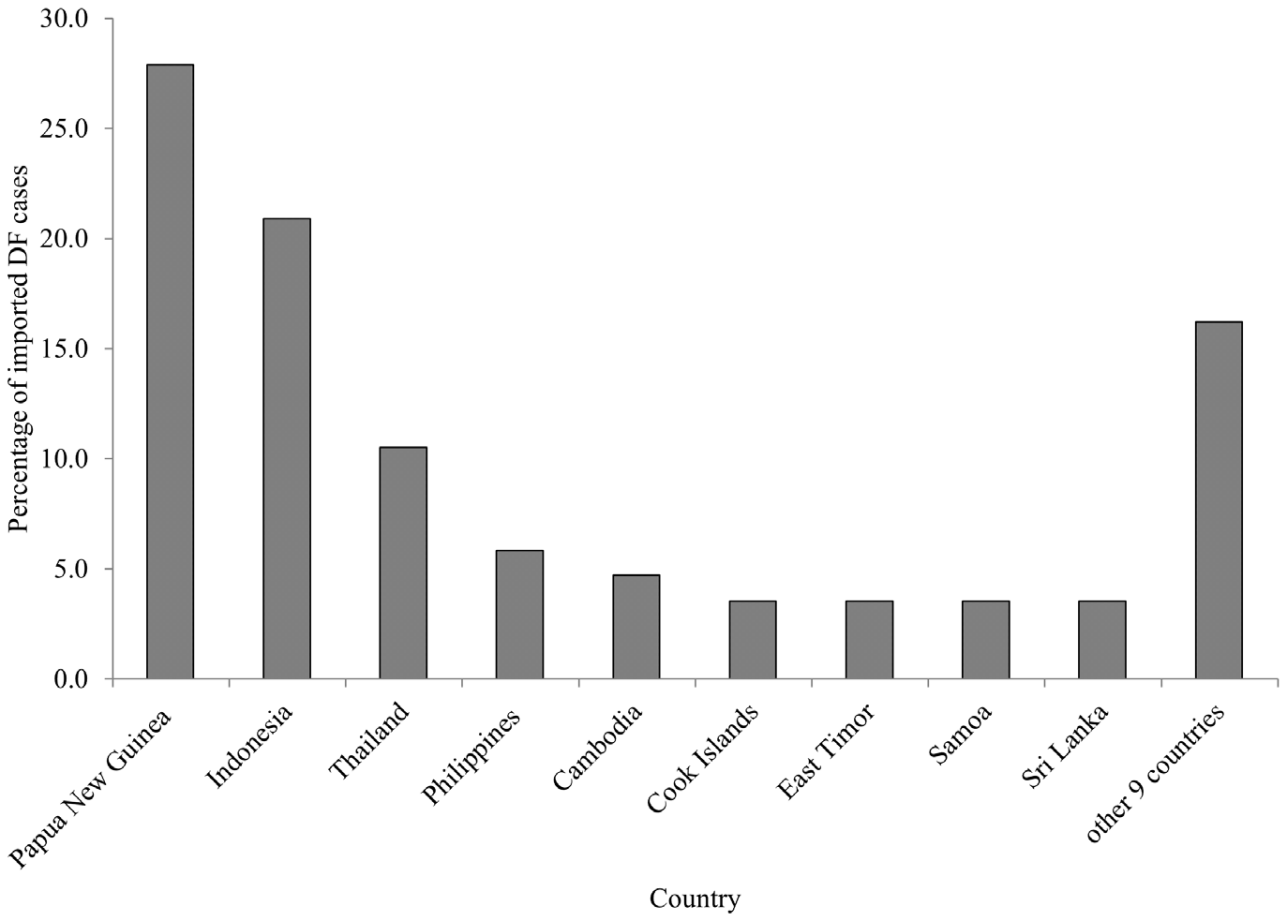
The percentages of imported DF cases from overseas between 1^st^ January 2000 and 31^st^ December 2009 in Cairns, Australia.

**Figure 2:**
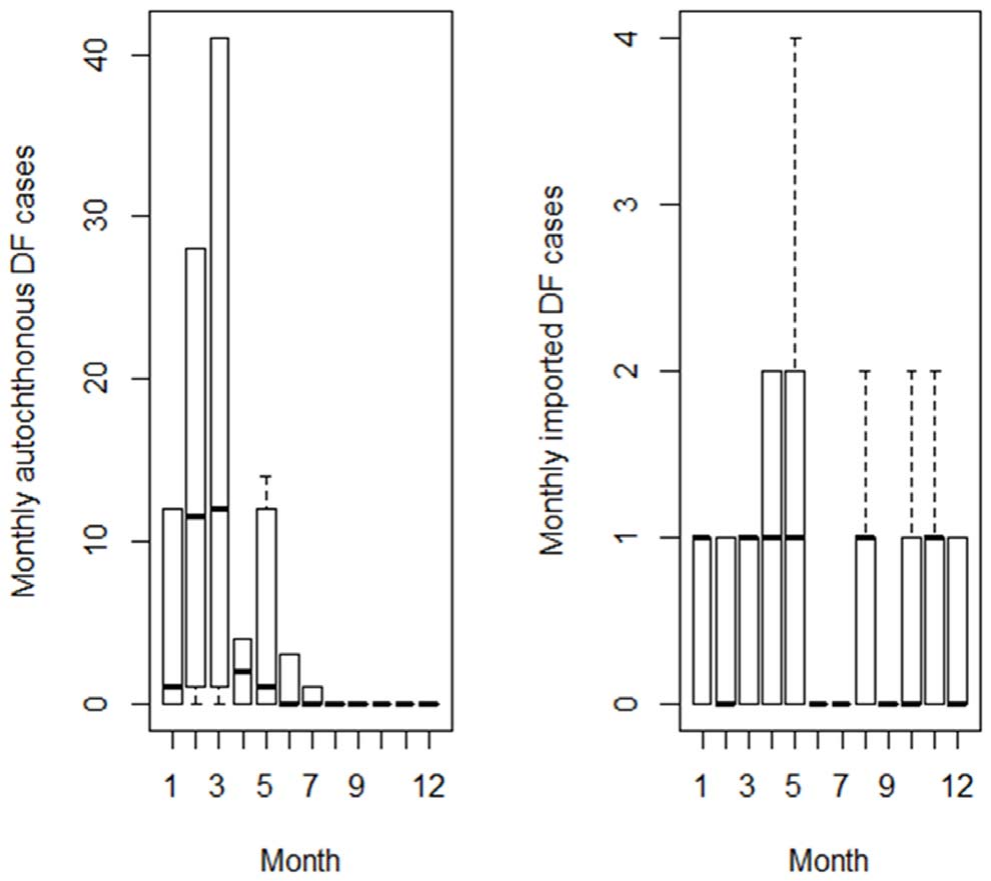
Boxplots of the numbers of autochthonous DF cases and imported DF cases for each month across the years 2000 to 2009 in Cairns, Australia.

During 2000–2009, there were total of 86 imported DF cases and 1865 autochthonous DF cases in Cairns. The countries of origin associated with the largest total numbers of imported DF cases were Papua New Guinea (27.9%), Indonesia (20.9%), Thailand (10.5%), Philippines (5.8%) and Cambodia (4.7%) in the study period (Figure 1). However, the total number of imported DF cases from Indonesia was larger than that of Papua New Guinea between 2004 and 2009. Figure 2 showed a strong seasonal epidemic pattern of the number of monthly autochthonous DF cases during the study period. The largest numbers of monthly imported DF cases were reported in April and May (Figure 2), although seasonal patterns were not as clear as for autochthonous DF cases.

**Figure 3:**
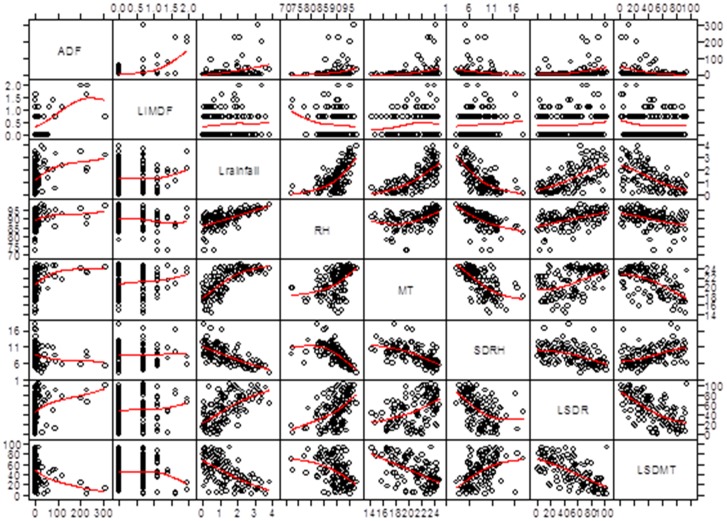
Pairwise scatterplot of monthly autochthonous DF cases and selected explanatory variables. ADF is monthly autochthonous DF cases. LIMDF is log-transformed monthly imported DF cases. Lrainfall is log-transformed monthly rainfall. SDRH is monthly standard deviation of daily relative humidity. LSDR is log-transformed monthly standard deviation of daily rainfall at a lag of one month. LSDMT is monthly standard deviation of daily minimum temperature at a lag of one month.

Over the period 2000–2009, the mean monthly RH was 89.8%, ranging from 72.3–98.0%. The monthly rainfall ranged from 0.0–44.4 mm with an average of 5.5 mm. The mean monthly MT was 21°C and fluctuated between 14.3 and 24.9°C. The mean monthly SDRH, SDR and SDMT were 8.5% (range 3.1–17.9%), 10.3 mm (range 0.0–61.1mm) and 1.8°C (range 0.5–4.2°C.), respectively. The pairwise scatter plot with smoother spline depicts the crude relationships between all the selected variables (Figure 3).

**Figure 4:**
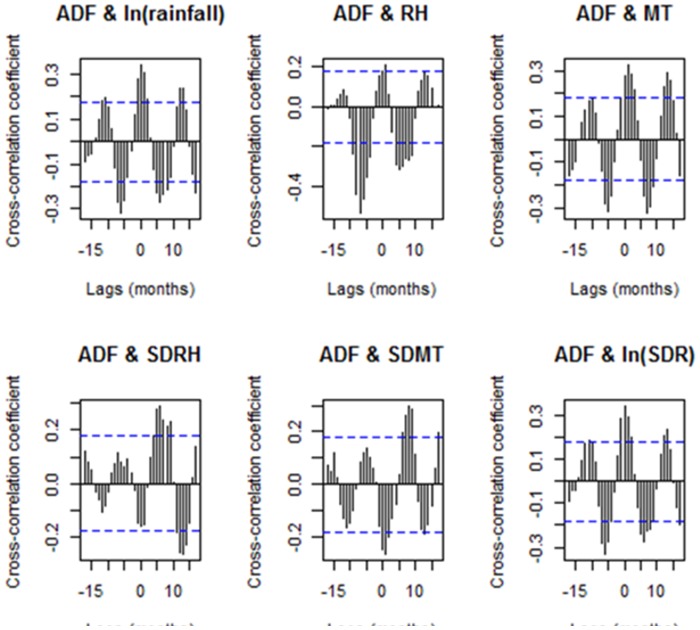
Plots of cross-correlations between number of monthly autochthonous DF cases and monthly climate variables during 2000 to 2009 in Cairns, Australia. ADF is monthly autochthonous DF cases. SDRH is monthly standard deviation of daily relative humidity. SDMT is monthly standard deviation of daily minimum temperature. ln(SDR) is log-transformed monthly standard deviation of daily rainfall. The two dash lines are critical values for cross-correlation (at the 5% level).

Lagged weather effects were identified by cross-correlation analysis. Figure 4 shows the cross-correlations between monthly autochthonous DF cases and selected climate variables. Significant cross-correlations at lags of 0–1 and 5–7 months were observed among log-transformed rainfall, MT and log-transformed SDR. Autochthonous DF cases were positively associated with RH at a lag of 0 months, and were negatively associated with RH at lags of 4–9 months. Autochthonous DF cases were negatively associated with SDMT at a lag of zero month. No lagged effects were observed between SDRH and autochthonous DF cases. We only included weather variables at lags of 0 or 1 month in the TNBH model because of DF biologic plausibility. The effects of lag of 4–9 months were considered as a seasonal variation.

### Time Series Hurdle Model

In this study, 60% of the observations on monthly autochthonous DF cases were zero values. The mean and variance of the numbers of monthly autochthonous DF cases were 15.5 and 2221, respectively. The data exhibited both over-dispersion and excess zeroes. Our results indicated that the TNBH model was flexible and suitable to our data based on AIC and Vuong non-nested test.

The selected variables and the final TNBH model were assessed by cross-correlation functions, the Vuong non-nested test and the AIC. The results indicated that the final TNBH model had the lowest AIC value (AIC = 421.12) in comparison with the time-series Poisson model (AIC = 817.9), the time-series negative binomial model (AIC = 442.9) and the time-series Poisson hurdle model (AIC = 695). The Vuong non-nested test also showed the superiority of the TNBH over the time-series negative binomial model (p<0.000). The residuals of the TNBH model appeared to fluctuate randomly around zero and with little autocorrelation after accounting for the AR(1) and AR(2).

Table 1 showed the estimated parameters, RR, OR and 95% CI for the negative binomial component and the zero hurdle component of the TNBH model. After adjustment for seasonality, population size and autocorrelation at lags of 1–2 months in the TNBH, the results of the negative binomial component showed that monthly autochthonous DF incidence was significantly and positively associated with log-transformed monthly imported DF cases (RR:1.52; 95% CI: 1.01–2.28), monthly MT (RR: 2.28; 95% CI: 1.77–2.93), monthly RH (RR: 1.21; 95% CI: 1.06–1.37), and monthly SDRH (RR: 1.27; 95% CI: 1.08–1.50). There was a statistically significant negative relationship between incidence of monthly autochthonous DF and log-transformed monthly rainfall (RR: 0.50; 95% CI: 0.31–0.81). Moreover, monthly MT (OR: 1.64; 95% CI: 1.01–2.67) was significantly related to the occurrence of monthly autochthonous DF cases in the zero hurdle model component.

**Table 1:** The estimated parameters, RR, OR and 95% confidence intervals for the count negative binomial portion and zero hurdle portion of the TNBH model.

Lag months	Parameter	Negative binomial partRR (95% CI)	Zero hurdle partOR (95% CI)
1	AR(1)	4.29 (3.18, 5.79)	7.25 (2.42, 21.69)
2	AR(2)	0.50 (0.39, 0.64)	0.64 (0.29, 1.44)
0	ln(importedcases)	1.52 (1.01, 2.28)	1.32 (0.36, 4.76)
0	ln(rainfall)(mm)	0.50 (0.31, 0.81)	1.75 (0.58, 5.30)
0	RH (%)	1.21 (1.06, 1.37)	1.04 (0.86, 1.25)
0	MT (°C)	2.28 (1.77, 2.93)	1.64 (1.01, 2.67)
0	SDRH (%)	1.27 (1.08, 1.50)	1.23 (0.90, 1.68)
1	ln(SDR) (mm)	1.09 (0.77, 1.54)	1.54 (0.65, 3.69)
1	SDMT (°C)	1.16 (0.59, 2.29)	0.58 (0.16, 2.12)

AR is autoregressive term. MT is monthly minimum temperature. SDRH is monthly standard deviation of daily relative humidity. ln(SDR) is log-transformed monthly standard deviation of daily rainfall. SDMT is monthly standard deviation of daily minimum temperature.

## Discussion

Our study concluded that observed incidence of autochthonous DF was significantly positively associated with the number of imported DF cases, but that the occurrence of autochthonous DF counts was associated with MT rather than the current number of imported DF cases. After accounting for incorporating imported DF cases, the study confirmed that local weather factors, including MT, rainfall and RH, strongly influenced the observed incidence of autochthonous DF in Cairns, Australia.

There was a significantly positive relationship between numbers of imported DF cases and autochthonous DF incidence in Cairns in the study. Approximately 90% of imported DF cases were from the countries in Pacific region and Southeast Asia. DF is endemic to countries in this region, including Papua New Guinea, Indonesia, Thailand, Philippines and Cambodia, which include popular holiday destinations for Australians, and where many Australians have family or work-related links. Our study indicated that imported DF cases strongly influenced autochthonous DF cases, and the occurrence of autochthonous DF was not associated with imported DF cases in the hurdle part. These findings could support the suggestion that imported DF cases play a significant role in influencing numbers of local DF cases under favourable weather conditions.

Our results revealed that MT and RH were significantly, positively associated with incidence of autochthonous DF. Temperature and RH have been demonstrated as the main determinants of DENV transmission and influence the life cycle of *Ae. aegypti*, like many previous studies [34]–[37]. Increasing temperatures lead to an increase in feeding frequency and shorter gonotrophic cycle, pupae development period, virus replication and extrinsic incubation period for *Ae. aegypti* [38]–[40], and thus enhance the risk of DF outbreaks. Increasing RH also favours oviposition, blood-feeding, longevity, virus propagation and density of *Ae. aegypti* [41]–[43].

Rainfall was also found to be negatively associated with autochthonous DF incidence in the study. Many researches have indicated that increased rainfall can increase the number of outdoor larval breeding places of *Ae. aegypti* and can therefore increase the density of mosquitos and enhance the risk of DENV transmission [40], [41], [44]. On the other hand, some studies have reported a negative relationship between rainfall and DF cases, which has been explained by larvae and eggs being washed away by massive rainfall [45]–[48]. Previous research indicated that many container types were suitable to *Ae. Aegypti* breeding sites in wet season in Cairns, such as pot plant base, pot plant pot, bucket, black plastic, small plastic container, palm frond and bromeliad, and that limited resources in containers significantly negatively influenced the abundance and size of *Ae. aegypti* in Cairns [49]. Cairns typically has a tropical monsoon climate with abundant amounts of rainfall in the form of frequent thunderstorms in the wet season [50]. Here, the result could be explained that heavy rainfalls might immediately wash away larvae from some subterranean sites and small, shallow containers. Moreover, frequent thunderstorms might flush away some resources from containers to lead to restriction of growth and development of larvae and reduction of the adult population of *Ae. aegypti* in Cairns.

Our result revealed that within-month variability in daily RH played a significant positive role in autochthonous DF incidence in Cairns. In general, RH variation is highly dependent on the fluctuations of temperature, precipitation and evaporation. Therefore, RH variation might be derived by the fluctuations of other weather factors, particularly heavy rainfall. Our study suggested that SDRH could be considered as a small-scale climate index to capture within-month climate variation, particularly in extreme weather events within a month in Cairns.

The study had several known limitations. First, underreporting would have been likely if people infected by DF had subclinical conditions and did not seek medical attention. Misclassification among the imported, autochthonous DF cases and other arboviral infections (eg., chikungunya) could also be possible. Second, a number of potential confounders (ie., virus strain, mosquito population densities and survival, population immunity and social factors) may affect the assessment of the relationship between weather variability and the DENV transmission. More detailed risk assessment may also be required. Additionally, previous study showed that the key determinants of observed DF incidence included temperature, rainfall and humidity. However, the advantage of our study was that the relationship between weather variables and autochthonous DF incidence was examined under accounting for imported DF cases and excess zeroes.

In summary, our research showed that monthly imported DF cases, local weather variables and inter-month relative humidity variability were strongly associated with incidence of monthly autochthonous DF in Cairns between January 2000 and December 2009. Imported DF cases might be a key driver in wet season. The result also indicated that increasing monthly RH fluctuation enhanced the risk of DENV transmission in Cairns. The results could provide new insights for the future control, prediction and prevention of the risk of DENV transmission. Local health authorities may give a close surveillance of imported DF cases and control local *Ae. aegypti* during favourable weather conditions for DENV transmission.
